# 半乳凝素-3在肺癌微环境中的免疫调控和治疗突破

**DOI:** 10.3779/j.issn.1009-3419.2025.101.11

**Published:** 2025-07-20

**Authors:** Hongbao ZHU, Jiong DENG, Tong WANG

**Affiliations:** ^1^261053 潍坊，山东第二医科大学基础医学院; ^1^School of Basic Medical Sciences, Shandong Second Medical University, Weifang 261053, China; ^2^256699 滨州，滨州医学院附属医院; ^2^Affiliated Hospital of Binzhou Medical College, Binzhou 256699, China

**Keywords:** 半乳凝素-3, 肺肿瘤, 肿瘤微环境, 免疫细胞, 肿瘤细胞, Galectin-3, Lung neoplasms, Tumor microenvironment, Immune cells, Tumor cells

## Abstract

肺癌是全球范围内发病率和死亡率最高的恶性肿瘤之一，其5年生存率长期处于较低水平，主要归因于早期诊断困难、肿瘤异质性高以及治疗抵抗性强等挑战。尽管近年来靶向治疗和免疫检查点抑制剂的应用显著改善了部分患者的预后，但多数患者仍面临原发性或继发性耐药问题。半乳凝素-3是一种在肺组织中广泛表达的多功能糖结合蛋白，包括支气管和肺泡上皮细胞、肺血管系统和免疫细胞，其通过调控肿瘤细胞增殖、免疫逃逸和血管生成，在肺癌进展中发挥核心作用。而在肿瘤微环境（tumor microenvironment, TME）中复杂的免疫抑制机制，不仅促进肿瘤生长和转移，癌症免疫疗法的功效部分也受到TME中存在的多种免疫抑制机制的限制，需探索新的调控靶点以突破治疗瓶颈。本综述系统解析了半乳凝素-3在TME中与免疫细胞（如T细胞、巨噬细胞）的相互作用机制，并评估了其作为治疗靶点的潜力（如抑制剂开发、联合免疫治疗），旨在为半乳凝素-3作为肺癌治疗新靶点的深入研究提供理论依据，并为克服当前免疫治疗耐药性提供新思路。

## Abstract (en)

Lung cancer remains one of the most prevalent and deadly malignancies worldwide, with persistently low five-year survival rates. This poor prognosis is primarily attributed to challenges such as difficulties in early diagnosis, high tumor heterogeneity, and strong therapeutic resistance. Although recent advances in targeted therapies and immune checkpoint inhibitors have significantly improved the prognosis of some patients, the majority still encounter primary or secondary resistance. Galectin-3, a multifunctional glycan-binding protein, is constitutively expressed in pulmonary tissues. Its expression encompasses bronchial and alveolar epithelial cells, the pulmonary vasculature, and resident immune cells. Galectin-3 plays a central role in lung cancer progression by regulating tumor cell proliferation, immune evasion, and angiogenesis. The complex immunosuppressive mechanisms within the tumor microenvironment not only facilitate tumor growth and metastasis but also partially limit the efficacy of cancer immunotherapies. Overcoming these barriers requires the exploration of novel regulatory targets to break through therapeutic bottlenecks. This review systematically elucidates the mechanisms by which galectin-3 interacts with immune cells (*e.g.*, T cells, macrophages) in the tumor microenvironment and evaluates its potential as a therapeutic target, including inhibitor development and combination immunotherapy strategies. The findings aim to provide a theoretical foundation for advancing galectin-3 as a novel therapeutic target in lung cancer and offer new perspectives for overcoming current immunotherapy resistance.

肿瘤微环境（tumor microenvironment, TME）作为肿瘤细胞与宿主免疫系统、基质成分相互作用的动态网络，包括肿瘤细胞、多种免疫细胞、细胞外基质以及细胞因子、趋化因子等可溶性分子，在肺癌进展和免疫逃逸中扮演着关键角色。在肺癌中，半乳凝素-3的高表达与肿瘤侵袭性增强、免疫抑制性微环境形成及不良预后密切相关^[1]^。半乳凝素-3是β-半乳糖苷结合蛋白家族的成员，在参与炎症反应、调节组织纤维化以及肿瘤发生发展中扮演关键角色^[2,3]^。半乳凝素-3在肺癌、结肠癌^[4]^、乳腺癌^[5]^、卵巢癌^[6]^等多种恶性肿瘤中高表达，并且可通过调节肿瘤细胞黏附、增殖、凋亡及免疫细胞功能，直接或间接影响肿瘤生物学行为。半乳凝素-3与TME各种成分之间相互作用，共同影响肿瘤的发生发展、转移和治疗效果。

## 1 半乳凝素-3与肺癌临床关联

半乳凝素-3是动物凝集素家族中唯一的嵌合型成员，其独特的结构特征使半乳凝素-3能够通过细胞内、外不同机制参与肿瘤发生发展，尤其在肺癌中展现出显著的临床相关性。肺癌作为全球癌症相关死亡的首要病因，其组织学亚型与分子特征显著影响治疗策略及预后。研究^[7,8]^表明，半乳凝素-3的表达水平在非小细胞肺癌（non-small cell lung cancer, NSCLC）中显著升高（腺癌：约70%病例呈弥漫性强阳性；鳞癌：约50%病例呈局灶性中等阳性），而在小细胞肺癌（small cell lung cancer, SCLC）中几乎不表达，提示其可能作为NSCLC特异性分子标志物。在NSCLC中，肺腺癌的特征为中至高度的半乳凝素-3表达，该表达主要见于癌细胞的细胞质和细胞核，且不同分化水平之间无明显差异。但在肺鳞癌中，半乳凝素-3的中至高度表达与高分化癌之间呈正相关^[7]^。

研究^[1,9]^发现血液中循环半乳凝素-3高水平可预测免疫检查点阻断治疗晚期或转移性NSCLC患者的预后不良。值得注意的是，血清半乳凝素-3水平在转移性肺癌患者中较局限性肿瘤升高^[10]^。进一步研究^[11]^揭示，半乳凝素-3的表达与NSCLC侵袭性密切相关。原发肿瘤中半乳凝素-3低表达水平还可以预测早期肺腺癌转移且与无复发生存率降低显著相关^[12]^。在早期的浸润性肺腺癌中，半乳凝素-3是预测肿瘤根治术后复发的潜在生物标志物^[13]^，这些发现凸显了半乳凝素-3在肺癌分型及预后评估中的潜在价值。

综上，对半乳凝素-3的靶向治疗是合理的。利用半乳凝素-3抑制剂如天然多糖（果胶）、碳水化合物、半乳凝素-3C、多肽、特异性单克隆抗体和核酸药物来调节半乳凝素-3介导的细胞黏附，抑制血管生成和肿瘤转移等^[14]^。果胶一方面直接降低半乳凝素-3的表达，另一方面通过某些信号途径调节细胞因子的表达，达到治疗疾病的效果^[15]^。在Wang等^[16]^的研究中，在缺氧微环境中使用富含半乳糖多糖处理后，通过抑制半乳凝素-3的表达来抑制葡萄糖转运蛋白-1的表达和葡萄糖吸收，从而降低细胞中的活性氧水平。除此之外，这种多糖还可抑制STAT3磷酸化来抑制肿瘤相关巨噬细胞（tumor-associated macrophage, TAM）极化。

基于半乳凝素-3对免疫微环境的调节作用，其抑制剂与免疫治疗的协同效应成为研究热点。免疫检查点抑制剂（immune checkpoint inhibitors, ICIs）在肺癌治疗中已取得了一定进展，但患者的响应率仍有限。半乳凝素-3可通过调节TME中的免疫抑制机制，影响ICIs的疗效^[17]^。有研究^[18]^发现，半乳凝素-3增加了肺癌细胞中程序性死亡配体1（programmed death ligand 1, PD-L1）的表达，而半乳凝素-3抑制剂作为佐剂，可增强PD-L1阻滞剂的抗肿瘤活性。此外，半乳凝素-3抑制剂与其他抗肿瘤药物（如化疗药物、靶向药物）联合应用，可发挥协同抗肿瘤作用，但化疗药物对不同病理类型的肿瘤细胞的作用不同，联合免疫治疗的疗效会不同^[19]^。

上述证据表明，半乳凝素-3通过驱动肺癌细胞恶性表型与微环境重塑的双重机制，成为肺癌进展的关键调控因子。据此，靶向半乳凝素-3的联合疗法（如抑制免疫逃逸、增强化疗敏感性）为肺癌治疗提供了理论依据。

## 2 半乳凝素-3对免疫细胞调控机制

TME是由肿瘤细胞、免疫细胞、基质细胞及细胞外基质等共同构成的动态网络，其免疫抑制特性是肺癌进展和治疗抵抗的核心驱动因素^[20]^。半乳凝素-3通过调控免疫细胞功能、重塑肿瘤细胞生物学行为及促进血管生成，在TME中发挥多重作用。本文重点关注半乳凝素-3对TME中具有抗肿瘤作用的淋巴细胞和巨噬细胞的影响。

### 2.1 T细胞耗竭——凋亡与功能抑制的双重路径

在TME中半乳凝素-3通过多种机制介导T淋巴细胞耗竭，但其调控效果在肺癌亚型之间存在显著差异。半乳凝素-3通过胞内、外双重机制抑制抗肿瘤T细胞活性。胞外半乳凝素-3通过结合T细胞表面CD45分子的N-连接和/或O-连接糖残基（含β-半乳糖苷）触发T细胞凋亡信号。除此之外半乳凝素-3与T细胞受体（T cell receptor, TCR）上的β-半乳糖苷特异性结合，干扰TCR的聚集与激活，显著削弱CD8^+^ T细胞的细胞毒性功能^[21,22]^。胞内半乳凝素-3在活化T细胞内表达，通过结合B细胞淋巴瘤-2（B-cell lymphoma 2, Bcl-2）抑制凋亡，但同时也可能促进程序性死亡受体1（programmed cell death protein 1, PD-1）上调的耗竭表型增强^[22]^。

T细胞的招募需要干扰素γ（interferon γ, IFN-γ）诱导的C-X-C趋化因子配体（C-X-C motif chemokine ligand, CXCL）9/10/11的趋化梯度。肿瘤分泌的半乳凝素-3可与糖基化的IFNγ和肿瘤细胞外基质中的糖蛋白结合，从而避免IFNγ扩散和形成IFNγ诱导的趋化因子梯度，影响CD8^+ ^T细胞募集^[23]^。该机制在半乳凝素-3高表达的NSCLC（尤其是腺癌）中更为显著，而在半乳凝素-3低表达的SCLC中影响较小^[14]^。半乳凝素拮抗剂增加了肿瘤内IFNγ的扩散、CXCL9梯度和针对肿瘤抗原的人CD8^+ ^T细胞的肿瘤募集。除此之外，肺腺癌中半乳凝素-3高表达者有更多的调节性T细胞（regulatory T cells, Tregs），提示半乳凝素-3参与招募Tregs调节免疫抑制^[1]^。

淋巴细胞激活基因-3（lymphocyte activation gene-3, LAG-3）是半乳凝素-3的细胞表面受体，两者直接结合抑制抗原特异性CD8^+^ T细胞，通过阻断信号激活抑制其杀伤活性。值得注意的是，半乳凝素-3对CD45和TCR的靶向作用具有糖链依赖性，O-连接糖残基介导凋亡信号，而N-连接糖链参与抑制T细胞迁移^[24,25]^。这些机制共同导致T细胞功能耗竭，促进肿瘤免疫逃逸。此外，半乳凝素-3与LAG-3的结合揭示了其通过非经典免疫检查点调控T细胞活性的新途径，为开发双靶点抑制剂提供了理论依据。

T细胞免疫球蛋白和含黏蛋白分子3（T cell immunoglobulin and mucin domain-containing protein 3, TIM-3）是肺组织中的重要调节因子。TIM-3表达于肺源性骨髓细胞中，半乳凝素-3通过上调STAT3的磷酸化来增加PD-L1的表达，协同经典免疫检查点介导T细胞耗竭^[18]^。此外，TIM-3通过半乳凝素-3介导小鼠肺组织髓系细胞的炎症反应，是炎症相关肺部疾病的治疗靶点^[26]^。

半乳凝素-3是T细胞功能耗竭的核心驱动因子，通过糖结合调控T细胞的调亡和招募，重塑肿瘤免疫微环境，诱导免疫抑制和免疫逃逸。在肺腺癌中，半乳凝素-3主要通过LAG-3受体介导CD8^+ ^T细胞的耗竭，促进肺癌进展。而鳞癌中，TIM-3的依赖性抑制作用更显著，这种差异可能与亚型特异性受体表达谱相关^[14]^。

### 2.2 巨噬细胞极化——从抗肿瘤M1到促瘤M2的转化

肺组织本身驻留巨噬细胞和募集来源的巨噬细胞均可以作为构成促进肿瘤生长发育的TAM（M2）^[27]^。微环境中的白细胞介素（interleukine, IL）-4、IL-13等介导M2巨噬细胞活化，随后激活半乳凝素-3和其他表型M2激活标志物^[22]^。半乳凝素-3结合CD98/β1整合素，激活PI3K通路，进一步强化M2表型^[28]^。半乳凝素-3是髓系细胞触发受体2（triggering receptor expressed on myeloid cells 2, TREM2）的潜在配体。随后大量的TREM2^+^巨噬细胞通过CXCL2与其受体趋化轴在肿瘤内募集。半乳凝素-3可抑制TREM2介导的巨噬细胞吞噬功能，促进TREM2^+^巨噬细胞（M1）向免疫抑制的TAM（M2）转化，在体内减弱的抗原提呈和共刺激功能，重塑肿瘤免疫微环境^[29]^。

在缺氧微环境中，半乳凝素-3的高表达对巨噬细胞M2极化有重要作用。水解柑橘果胶因富含半乳糖对巨噬细胞的功能有显著影响。该多糖不仅可抑制半乳凝素-3的表达，下调葡萄糖转运蛋白-1的表达和葡萄糖摄取，从而降低细胞内活性氧水平，还可抑制STAT3的磷酸化，抑制巨噬细胞M2极化^[16]^。Kirsten大鼠肉瘤病毒癌基因同源物（Kirsten rat sarcoma viral oncogene homolog, *KRAS*）突变的肺腺癌细胞表现出对巨胞饮作用的显著依赖。其分子机制在于αvβ3整合素、KRAS蛋白及半乳凝素-3三者形成的信号复合物，该复合物是激活并驱动巨胞饮过程的关键^[30,31]^。此外，半乳凝素-3通过阻断集落刺激因子-1受体来耗竭巨噬细胞。因此，利用PD-1拮抗剂，改善T细胞浸润和抗肿瘤活性，抑制TAM活性，可提高免疫检查点治疗的有效性^[32]^。

半乳凝素-3是巨噬细胞M2极化特异性或上调标志物，但其作用在不同肺癌亚型中呈现显著差异。在NSCLC特别是腺癌中，IL-4等介导的半乳凝素-3高表达正向调控巨噬细胞环路活性；而在SCLC中，半乳凝素-3缺失表达^[7,28]^。NSCLC中，半乳凝素-3介导的TAM可通过激活Notch1信号，激活内皮细胞，促进肿瘤血管形成^[14]^。在临床上，抑制半乳凝素-3可阻断M2巨噬细胞极化，这有利于开发基于肺癌亚型的半乳凝素-3抑制治疗策略。

### 2.3 其他免疫细胞——免疫逃逸的协同作用

半乳凝素-3抑制自然杀伤（natural killer, NK）细胞介导的肿瘤免疫反应，半乳凝素-3降低I类主要组织相容性复合体相关链A（MHC class I chain-related protein A, MICA）对NKG2D（NK激活受体）的亲和力，并作为NKp30（NK激活受体）的抑制性配体，抑制NK细胞的细胞毒性^[33]^。有研究^[34]^发现，IL-3致敏的嗜碱性粒细胞在与肿瘤细胞半乳凝素-3与IgE结合后，激活分泌IL-4/IL-13，促进肿瘤细胞释放IL-6和血管内皮生长因子，加速血管生成从而促进肿瘤进程。

## 3 半乳凝素-3促进肿瘤进展分子网络

### 3.1 参与细胞间黏附

半乳凝素-3可与肿瘤组织中多种蛋白质相互作用，包括细胞外基质中蛋白以及肿瘤细胞表面蛋白。在肺腺癌中，内源性半乳凝素-3的表达抑制，可减少肿瘤细胞与肺血管内皮细胞的黏附，减少细胞的侵袭和迁移。外源性和内源性半乳凝素-3均通过增加核内β-catenin的积累而增加N-钙黏蛋白（N-cadherin, N-cad）和CD44的表达，从而促进低表达跨膜黏蛋白1的癌细胞与血管内皮的黏附^[35]^。此外，糖蛋白VI（glycoprotein VI, GPVI）是一种血小板特异性的胶原和纤维蛋白受体，通过识别半乳凝素-3协同发挥作用。血小板GPVI可与肿瘤细胞表达的半乳凝素-3之间发生相互作用，半乳凝素-3可激活血小板中的免疫受体酪氨酸激活基序（immunoreceptor tyrosine-based activation motif, ITAM）信号，增强血小板与癌细胞黏附，利于肿瘤细胞外渗，促进癌转移^[36]^。

半乳凝素-3结合蛋白（galectin-3-binding protein, LGALS3BP）表达在肿瘤细胞表面，是半乳凝素-3的天然配体。LGALS3BP通过与邻近细胞表面的半乳凝素-3残基相结合，诱导同源细胞间的黏附，促进癌栓形成，增强肿瘤细胞转移性^[37]^。值得注意的是，该机制在转移性肺腺癌中显著增强。

### 3.2 促进肿瘤细胞增殖

肿瘤干细胞（cancer stem cells, CSCs）是肿瘤形成的起始细胞，在肿瘤形成、生长等过程中起决定作用，具有使肿瘤无限增殖的潜能。半乳凝素-3通过EGFR/c-Myc/Sox2轴增强肺腺癌细胞的干性表达^[38]^。此外，不论在NSCLC原发或者转移部位，半乳凝素-3可通过抑制糖原合成酶激酶-3β，增加核内β-catenin，上调CD133等干细胞标志物^[39]^。

不仅如此，半乳凝素-3作为Toll样受体4（Toll-like receptor 4, TLR4）的配体，诱导肺腺癌细胞TLR4信号的激活，从而激活下游p65核转位，激活核因子κB（nuclear factor kappa-B, NF-κB）通路，促进核内长链非编码RNA的表达，最终影响肺腺癌细胞的增殖和迁移^[40]^。有研究^[30,41]^设计了一种“局部氧化-偶联”的糖链编辑方法，特异性阻断avβ3整合素与半乳凝素-3结合，抑制Kras驱动的巨噬细胞大胞饮作用和下游效应物的激活，并减轻Kras驱动的恶性表型。

### 3.3 调节肿瘤细胞调亡

半乳凝素-3对不同细胞的抗（促）凋亡作用取决于亚细胞类型和刺激的性质^[42]^。半乳凝素-3可通过抑制线粒体调亡通路抑制细胞凋亡，这也是NSCLC化疗抵抗的关键机制^[22,43]^。丝裂原活化蛋白激酶（mitogen-activated protein kinase, MAPK）在调节细胞生长、分化、凋亡及耐药性方面发挥重要作用。胞外半乳凝素-3能通过下调细胞外信号调节激酶（extracellular signal-regulated kinase, ERK）、应激激活蛋白激酶（c-Jun N-terminal kinase, JNK）的表达进一步抑制MAPK信号通路，发挥抗细胞凋亡的作用^[44]^。半乳凝素-3是肺纤维化的潜在机制。Bruceine A（BA）是一种含多种药理作用的化合物，可以靶向半乳凝素-3，干扰半乳凝素-3与转化生长因子-β1（transforming growth factor-beta 1, TGF-β1）蛋白之间的相互作用，抑制下游TGF-β1/Smad通路，逆转肺纤维化过程^[45]^。推测该机制也适用于肺癌微环境。

### 3.4 诱导肿瘤细胞转移

上皮-间质转化（epithelial-mesenchymal transition, EMT）是肿瘤侵袭和转移的前提。其重要分子特征是E-钙黏蛋白（E-cadherin, E-cad）功能丧失以及N-cad和波形蛋白等功能增强，使肿瘤细胞特别是E-cad的表达减少，失去与邻近细胞的紧密连接，从而脱离原发肿瘤，同时获得间质细胞的运动特征，向肿瘤基质中迁徙和游走，促进肿瘤转移。

在肿瘤细胞中，内源性和外源性的半乳凝素-3都可以上调N-cad，促进肿瘤细胞与人血管内皮细胞的黏附，从而促进肿瘤的生长及转移^[35]^。在肺癌中，EMT与PD-L1的表达有关，PD-L1水平升高会抑制免疫系统，从而使癌症更容易扩散^[46]^。研究^[11,35]^发现NSCLC中半乳凝素-3通过激活Wnt/β-catenin通路，上调N-cad，同时抑制E-cad，驱动EMT。在香烟烟雾暴露的气道上皮细胞（airway epithelial cell, AEC）中，活性氧的产生增加，这可促进Runt相关转录因子2和半乳凝素-3的上调。后者通过下调上皮标志物并促进间充质标志物和干细胞标志物，影响AEC的EMT，导致AEC的侵袭、迁移、集落形成和肿瘤球形成^[47]^。血管生成是肿瘤细胞侵袭和转移的关键步骤。研究^[48]^表明半乳凝素-3可通过激活以Jagged1依赖的Notch信号通路为核心轴，促进肿瘤血管生成，从而驱动肿瘤转移。

与半乳凝素-3结合的LGALS3BP也可参与肺腺癌细胞侵袭。肺腺癌细胞外泌体来源的LGALS3BP与破骨细胞相互作用，一些关键破骨细胞标志物的调节失调，这可能有助于促进破骨细胞发生，参与肺腺癌骨转移^[49]^。微管组织中心蛋白2A（microtubule-organizing center protein 2A, MZT2A）调节细胞中的微管成核活性。MZT2A诱导AKT磷酸化，通过上调LGALS3BP，促进NSCLC增殖和侵袭，并且在选择性AKT抑制剂使用后可阻断这些作用^[50]^。此外，一些细胞因子如IL-6、粒细胞集落刺激因子等，也是肿瘤发生转移过程中的重要启动因子，促进晚期NSCLC转移微环境的形成。

## 4 小结与挑战

半乳凝素-3作为TME的核心调控分子，通过多种机制驱动肺癌进展。半乳凝素-3可通过诱导T细胞凋亡、促进M2型巨噬细胞极化，以及抑制NK细胞活性，显著削弱抗肿瘤免疫应答，重塑免疫抑制性TME。同时，半乳凝素-3影响肿瘤细胞黏附、增殖、凋亡、转移，驱动肿瘤恶性表型。目前半乳凝素-3在肺癌微环境中的作用机制尚未完全阐明，其亚细胞定位（核内/胞质）如何影响信号通路选择（如促凋亡或抗凋亡）仍需进一步验证。进一步揭示半乳凝素-3的生物学功能，优化其作为生物标志物的应用，开发更有效的半乳凝素-3抑制剂，并探索其在联合治疗中的最佳策略是未来值得进一步研究的方向。
